# Health systems guidance appraisal—a critical interpretive synthesis

**DOI:** 10.1186/s13012-016-0373-y

**Published:** 2016-01-22

**Authors:** Denis E. Ako-Arrey, Melissa C. Brouwers, John N. Lavis, Mita K. Giacomini, on behalf of the AGREE-HS Team

**Affiliations:** 1 McMaster University, Juravinski Hospital Site, G Wing, 2nd Floor, Room 207, 711 Concession Street, Hamilton, ON L8V 1C3 Canada; 2 McMaster University, MML-417, 1280 Main St. West, Hamilton, ON L8S 4L6 Canada; 3 McMaster University, CRL-218, 1280 Main Street West, Hamilton, ON L8S 4K1 Canada

**Keywords:** Health systems guidance, Guidance development, Guidance appraisal, Guidance reporting, Health systems challenges, Health systems arrangements, AGREE-HS

## Abstract

**Background:**

Health systems guidance (HSG) are systematically developed statements that assist with decisions about options for addressing health systems challenges, including related changes in health systems arrangements. However, the development, appraisal, and reporting of HSG poses unique conceptual and methodological challenges related to the varied types of evidence that are relevant, the complexity of health systems, and the pre-eminence of contextual factors. To address this gap, we are conducting a program of research that aims to create a tool to support the appraisal of HSG and further enhance HSG development and reporting. The focus of this paper was to conduct a knowledge synthesis of the published and grey literatures to determine quality criteria (concepts) relevant for this process.

**Methods:**

We applied a critical interpretive synthesis (CIS) approach to knowledge synthesis that enabled an iterative, flexible, and dynamic analysis of diverse bodies of literature in order to generate a candidate list of concepts that will constitute the foundational components of the HSG tool. Using our review questions as compasses, we were able to guide the search strategy to look for papers based on their potential relevance to HSG appraisal, development, and reporting. The search strategy included various electronic databases and sources, subject-specific journals, conference abstracts, research reports, book chapters, unpublished data, dissertations, and policy documents. Screening the papers and data extraction was completed independently and in duplicate, and a narrative approach to data synthesis was executed.

**Results:**

We identified 43 papers that met eligibility criteria. No existing review was found on this topic, and no HSG appraisal tool was identified. Over one third of the authors implicitly or explicitly identified the need for a high-quality tool aimed to systematically evaluate HSG and contribute to its development/reporting. We identified 30 concepts that may be relevant to the appraisal of HSG and were able to cluster them into three meaningful domains: process principles, content, and context principles.

**Conclusions:**

Our study showed the role that the quality criteria play in the development, appraisal, and reporting of HSG and demonstrated the link and resonance within and between the various concepts in the three domains.

## Background

Roemer [1] defined a health system as the combination of resources, organizations, and financing and management bodies that culminate in the delivery of health services to a population. A redefinition of the term health system was proposed by the World Health Organization [2] to consist of all organizations, people, actions, and activities whose primary purpose is to promote, restore, and maintain health. This was further modified—to be more comprehensive and explicit—by a group convened by the World Health Organization’s (WHO) Alliance for Health Policy and Systems Research, to refer to delivery and financial and governance arrangements for health care and population health services and the broader context in which they are negotiated, implemented, and reformed [3].

Health systems guidance (HSG) are systematically developed statements produced at global, national, and regional levels to assist with decisions about appropriate options for addressing health systems challenges (including related changes in health systems arrangements), the implementation of these options, and the monitoring and evaluation of the implementation efforts [4]. The need to develop and use evidence-informed approaches to address national and global health system issues through the use of HSG has been articulated by many [5]. For example, the Millennium Development Goals (MDGs) reflect eight international goals (e.g., reduce child mortality, improve maternal health, and combat HIV/AIDS, malaria, and other diseases) and were developed by the United Nations in 2000. However, their attainment has been hindered by weak health systems and lack of system-specific recommendations [6, 7]. Formulating recommendations to address root causes has the potential to clear the path toward better health outcomes in line with health goals [8].

The terms *guidelines* and *guidance* both refer to any document containing a recommendation on a course of action. However, in contrast to the word “*guidelines*,” the term “*guidance*” is used in the health system context in order to make explicit the difference between the process of supporting evidence-informed judgments for health system issues from that of clinical judgments as would be expected with clinical practice guidelines (CPGs). As a policy-oriented product, HSG represents the whole body of knowledge that informs policy decisions on how health system issues should be classified or prioritized, appropriate health system governance, optimal financial arrangements, system organization, and the design and delivery of effective health programs and services [9–12].

The main bodies tasked with developing HSG are international and intergovernmental organizations (e.g., WHO, Pan American Health Organization), local ministries of health, and special national committees or agencies providing support to ministries of health. Their production is usually linked with these high-level health sector entities and less frequently in decentralized structures at the sub-national level. HSG may be aligned with specific goal-oriented taxonomies (e.g., WHO building blocks), unique national/regional governance, or tailored to financial/delivery arrangements with the common goal to optimize health care systems. However, while these organizations have demonstrated an interest in the development, appraisal, and reporting of this type of guidance as described in the WHO’s Handbook for Supporting the Development of Health Systems Guidance [13], for example, many of their members and collaborators (who are also members of the Appraisal of Guidelines for Research and Evaluation for Health Systems (AGREE-HS) team) contend that these intensions have not been matched by adequate action or results, in part, due to the lack of experience in developing HSG.

Indeed, in comparison to CPGs, the development of HSG pose different conceptual and methodological challenges related to the varied types of evidence that are relevant, valued, and considered; the complexity of health systems’ internal and external relations that must be addressed; and the pre-eminence of contextual factors that directly influence the design and adoptability of recommendations [4]. As with CPGs, HSG statements or recommendations should be justified by assessments of the quality of evidence supporting them, the potential for unintended consequences, and by discussions of implementation and contextual issues. For example, the full implementation of HSG recommendations can be further hampered by “bottlenecks” like health system fragmentation and capacity limitations; these limitations should also be addressed in the guidance documents [14, 15].

The potential for positive impacts by HSG as a decision tool to improve health systems is great. However, as with CPGs [16], this potential is only as good as the quality of the HSG. Indeed, the ability to impact and optimize health system performance and efficiency through the development and adoption of HSG is hampered by the dearth of tools to guide their development, appraisal, and reporting. As a consequence, this leaves knowledge users at a loss when choosing the highest quality and/or most appropriate guidance or in creating new guidance in circumstances where there is none or navigating circumstances where the existing guidance is not credible or of poor quality. In contrast to the development, appraisal, and reporting methods for CPGs, the development of HSG is still at a rudimentary stage [4]. There has been some work related to the use of “evidence briefs” to assist policymakers and stakeholders with working through a health system problem, options for addressing it, and key implementation considerations, informed by the best available data and research evidence [17]. However, there is a need for systematically and transparently developed guidance that can feed into such context-specific documents. With the rising trends that encourage bridging the gap between research and policy and practice, this is a significant research gap in both the science and practice of knowledge translation. HSG can provide this bridge between research synthesis and policy needs for evidence.

The creation of high-quality HSG requires tools to support the development and reporting of high-quality guidance and tools able to differentiate between high- and low-quality reports. There is also value in establishing acceptable quality thresholds and creating common methodologies and nomenclature among the HSG community (developers, users, and researchers). At present, however, there is no universally acceptable gold standard approach for appraising HSG, although there are some tools (for example the Handbook for Supporting the Development of Health System Guidance) to support their development [13] and reporting. To address these gaps, we are conducting a program of research with the international health systems guidance community that aims to create a tool to support the appraisal of HSG and further enhance HSG development and reporting.

The first step to this program of research, and the focus of this paper, was to conduct a knowledge synthesis of the published and grey literatures to determine concepts (items, criteria, or domains) related to HSG development, reporting, and quality. The results of the synthesis are to provide the foundational components of our HSG tool and to serve as a conceptual status report for the research community.

## Methods

With the goal of generating a list of concepts that can be used to develop, appraise, and report HSG, we conducted an initial search to identify existing concepts, tools, templates, or checklists that have been used or could be used to describe, differentiate, or appraise the quality of HSG. The aim of this initial search was to determine if reviews on this topic exist, estimate an initial number of relevant papers available, highlight additional and useful search terms, and clarify inclusion and exclusion criteria and the most appropriate knowledge synthesis approach [18, 19].

After contemplating the variety of knowledge synthesis approaches available, a critical interpretive synthesis (CIS) approach was considered the most appropriate for three reasons. First, CIS is a systematic approach that facilitates the analysis of complex and diverse bodies of literature including qualitative, quantitative, and theoretical papers [20–26]. The available literature on HSG is highly heterogeneous and methodologically diverse, comprising of a mix of empirical qualitative and quantitative papers and non-empirical papers. A wider range of evidence and study designs are typical occurrences in health systems/health policy research and HSG is a nascent domain where good thinking is likely to be captured in expert opinion/views, editorial comment, policy documents, political statements, experiences of stakeholders, theoretical/discussion papers, and other colloquial forms of evidence. In contrast to CIS, conventional systematic reviews, for example, have been criticized for excluding forms of evidence traditionally considered as non-experimental [20, 27–30].

Second, the objective of a CIS is to develop new concepts and theories through a typically interpretive mode of inquiry. This is in contrast to more conventional systematic review approaches where the mode of inquiry is more aggregative and aimed at testing theories by collating, compiling, pooling, and summarizing common outcomes across a range of studies [19–21, 25–27, 31, 32]. Since our review aims at generating a candidate list of items, criteria, or domains for HSG development, appraisal, and reporting, this theory-generating approach that CIS promotes was deemed a good fit.

Third, CIS offers a more flexible, iterative, dynamic, and reflective approach requiring investigators to assess the extent to which new information or data are provided with each additional paper considered. It applies a relatively loosely defined set of processes for critically analyzing and synthesizing literature [19, 20, 23, 27, 28, 30]. This differs from the more conventional systematic review methods which have clearly stated study protocols, an exhaustive search of all available literature, standardized data extraction templates, and explicit quality appraisal checklists, as well as pre-determined focused questions, strict inclusion and exclusion criteria, and specified data boundaries [20, 23, 27–30].

With the unique conceptual and methodological challenges related to the development of HSG [4], it was essential to have a review method that was iterative, flexible, and dynamic. Our review was not simply aimed at summarizing this complex literature on HSG, so we sought to produce a logical and insightful interpretation of a purposefully sampled body of evidence. As such, the aim was to yield a comprehensive interpretation without requiring exhaustive identification of relevant items of literature as in a conventional systematic review [20, 29, 30].

### Review question(s)

As per CIS methodological standards, our review questions served as compasses rather than anchors [20, 29, 33] allowing for the concepts of HSG to be derived from synthesis of the literature and constantly modifying them in an iterative manner throughout the review.

Our guiding review questions were as follows:

1. How have authors variously defined HSG quality in the literature?
2. How have authors interpreted and used criteria (or tools or instruments, checklists, systems, etc.): to describe or define HSG quality or reporting requirements, to appraise HSG quality, or to differentiate between HSG on the basis of quality?
3. What methods have authors and stakeholders used to develop these criteria?
4. What methods have authors and stakeholders used to address health system issues/challenges?

### Literature search

In searching the literature, our goal was to select papers based on their potential relevance to HSG appraisal and quality, while including other papers that, though not directly relevant to health systems, were deemed important for the purpose of our review. Instead of including an exhaustive number of papers, our plan was to provide a comprehensive sampling frame of potentially relevant papers using emergent eligibility criteria [25, 32]. As per the standards of a CIS, the boundaries of our inclusion and exclusion criteria were modifiable, dynamic, and continuously shifting [20, 32, 34]. The goal was to populate the concept of HSG with new concepts rather than finding papers that reiterated ideas already captured in previously reviewed papers.

### Eligibility criteria

1. Study content: (a) Papers that evaluated HSG or papers that report on criteria/tools that have been documented as important indicators of HSG quality and (b) papers that reported on methods for addressing health system issues/challenges.
2. Time frame: Papers are eligible if published in or after the year 2000 (when the first World Health Report on health systems [2] was published).
3. Context: Unrestricted. We sought papers that considered HSG evaluation and health system issues/challenges in various contexts (low-, middle-, and high-income countries).
4. Study design: Any study design.
5. Language: English, French, and Spanish

A combination of key and free text terms were used to search through various databases. Terms used were health systems, health policy, guidelines, guidance, health services arrangement, health services organization, health system issues, health system challenges, tools, instruments, criteria, items, domains, evaluation, appraisal, quality, and standards.

The search strategy included various electronic databases and sources: Cumulative Index to Nursing and Allied Health Literature (CINAHL), Cochrane Library, Excerpta Medica dataBASE (EMBASE), Google Scholar, Health Systems Evidence, Latin American and Caribbean Health Sciences Literature (LILACS), PubMed, Virtual Health Library, and Web of Science. We also searched subject-specific as well as regional electronic sources: Australia’s National Health and Medical Research Council, Evidence Best Practices for Public Health, University of Massachusetts National Guidelines Clearinghouses, National Institute of Health and Care Excellence (NICE), WHO’s Evidence Informed Policy Network (EVIPNet), WHO EURO’s Health Evidence Network, and Guidelines International Network (GIN) directories.

We also searched for other grey literature, including conference abstracts (Global Symposium on Health Systems Research, Canadian Association for Health Services and Policy Research, Health Systems and Process Improvement Conference, Canada’s Health Leadership Conference, Health Systems Reform in Asia Conference, International Society on Priorities in Health Care). Further, research reports, book chapters, unpublished data, dissertations, and policy documents that were nominated by members of the team or found in unique holdings of health sciences libraries in Canada were also included. Additional papers were identified by manually searching bibliographies, while more were obtained by hand searching some key journals (i.e., Health Policy and Planning, Health Services Research and Policy, Health Research Policy and Systems, Global Healthcare Systems, Health Systems and Reform, and Health Policy). This was a complementary search strategy to account for papers not included in electronic databases or with search terms that do not allow them to be easily identified [34, 35].

Finally, we also contacted (through emails, phone, Skype or in person) experts, colleagues, and members of our research team with a known interest on this topic to identify additional papers (published, unpublished, or ongoing). To increase the scope of these key informants, we also asked initial contacts to refer us to others who can provide more information. Relevant papers were then imported to Endnote bibliographic software.

A two-step sampling process was used. First, a purposeful sampling approach [36, 37] was used to investigate the literature to determine the range of unique candidate concepts—or theoretical domains—associated with HSG. We stopped sampling at the saturation point where looking at new literature no longer contributed additional concepts [25, 37]. Second, a theoretical sampling strategy was used to interrogate the literature relevant to each of the identified concepts. Theoretical sampling does not occur at a single point in the research process but is a recurrent feature, which aligns it well with the dynamic CIS methodology. Similar to the first approach, we stopped sampling at the saturation point where looking at new literature no longer contributed additional descriptions of the identified concepts. The intent here was to thoroughly capture the depth of the concept across the literature in order to generate and develop the theoretical underpinnings of the ideas rather than collecting numerous citations of identical concepts and/or descriptions. Screening titles, abstracts, and full text was completed independently and in duplicates (DAA and SA). Disagreements were resolved by consensus.

### Quality appraisal

We did not consider it appropriate to undertake quality appraisal for three principal reasons: firstly, the literature on HSG is diverse and complex; secondly, the available literature could not be hierarchically ordered in terms of study design, importance, or relevance; and thirdly, very little consensus exists on how or whether to perform quality appraisal in a CIS methodology [27, 32, 38, 39]. Instead, we chose to evaluate papers for inclusion based on a judgment of their relevance and likely contribution to concept development and theory [23, 26, 32, 40]. Our assumption was that some methodologically weak papers are theoretically and conceptually pertinent [25, 29, 34]. The goal of our review was to generate key concepts relevant to the appraisal of HSG, so we applied a flexible relevance boundary in order to be as inclusive and comprehensive as possible and include papers that could contribute to this theory generation.

### Data extraction and analysis

As a result of the nature of the data, we did not use a standardized data extraction template to retrieve the relevant elements of this review from all the retained papers. Instead, and as per CIS methodology, we gave latitude for a more narrative data retrieval approach. It was, however, possible to extract some key elements from all the papers including author(s), the year of publication, geographical location of the study or affiliation of the author, purpose of the study/paper, its relevance and a summary of its main conceptual contributions, and types of items, criteria, or domains considered. Data were extracted independently and in duplicate (DAA and SA) with disagreements resolved via consensus. The extracted data were then compiled in summary form.

Again due to the anticipated diversity of methodological designs and implementation of the different items, criteria, and domains that were extracted from the papers, a narrative approach to data synthesis was executed. The data extracted were sorted and categorized into groups with themes. We sought to collect similar concepts within the same group. This systematic method of recording themes, and making connections between themes and the data collected within a comprehensive category system [41, 42], is an advocated concept-development coding approach [43]. The information was imported into the analysis software NVivo version 9 in order to analyze groups of themes that depicted similar patterns in information. We did not organize the concepts in any hierarchical order but reported the concepts that were identified in each of the papers in this review.

Modeling from the health policy analysis triangle framework that was developed by Walt and Gilson [44], we were able to connect the concepts (constructs) together into meaningful domains. The framework considers all the essential elements that interact to shape policymaking by demonstrating that health policy should focus on the processes contingent on developing and implementing change, on the content of health policy reform, and on the context within which the policy is promulgated, as well as on the actors involved in the policy reform [44, 45]. The health policy analysis triangle framework is a highly simplified model of an extremely complex set of interrelationships between the different elements of the model (process, content, context, and actors) with each element influencing or being influenced by the other [45, 46]. Development, appraisal, and reporting of HSG play an important role in health policy making by providing options and recommendations to address a health systems issue. Therefore, similar to health policymaking, HSG development, appraisal, and reporting can be seen to occur in a series of discrete yet interconnected components of process, content, and context.

## Results and discussion

**Fig. 1:**
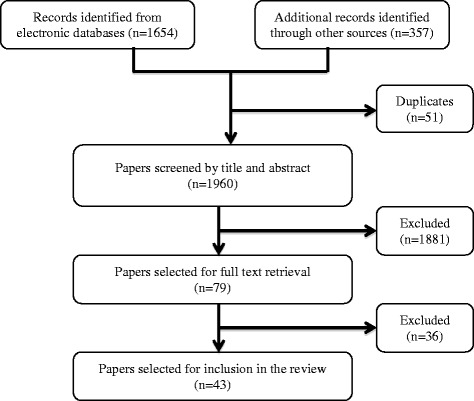
Flowchart of study selection

No existing knowledge synthesis was found on the topic, and no existing HSG appraisal tool (draft or final version) was identified. We identified a total of 43 papers that met our eligibility criteria and reported on concepts (items, criteria, domains) considered directly or conceptually relevant to HSG and/or their quality (see Fig. 1 for a flowchart of the study selection).

Forty-seven percent of the retained studies were technical reports, 32 % were concept papers, 13 % were quantitative studies, and 8 % used a mixed methods approach. The authors held affiliations at the following organizations: 33 % at universities, 25 % at the WHO, 23 % at research institutes, 12 % at government agencies (United Kingdom’s National Health Services [NHS], United States’ Centre for Disease Control [CDC], United States Agency for International Development [USAID], United Kingdom’s National Institute for Health and Care Excellence [NICE]), and 7 % at national ministries of health. Fifty-eight percent (58 %) of the papers were lead authored by an individual based in Europe, 30 % in North America, 8 % in Australia, 2 % in Asia, and 2 % in Africa. Over one third of the authors implicitly or explicitly identified the need for a high-quality tool aimed to systematically evaluate HSG and contribute to development and reporting of HSG. Thirty concepts were identified that are considered to be a good fit for, and may be relevant to, the development, appraisal, and reporting of HSG. The Appendix provides a description of the papers, their objectives, relevance to the HSG process, and the concepts extracted.

### Process principles, content, and context principles

Ostrom [47] stated that identifying key concepts and relationships among them culminates in the generation of a theory, which connects these key concepts. We were able to organize the concepts and identify relationships within and across them. Through an iterative process, we clustered the concepts together into three meaningful domains: process principles, content, and context principles.

#### Process principles

Process principles represent the methodological elements and the defining principles that demonstrate the development integrity of the HSG. They refer to the ways in which the HSG is initiated, developed, and formulated and can be looked upon as the “who” and “how” of the guidance [44, 46]. Here, we find the procedures and principles that were employed by the HSG developers in coming up with the guidance recommendations. Process principles are critical in the development of HSG because the methodological strategy is crucial for guidance appraisal and for differentiating across HSG of varying quality. They are also important because they can articulate the tactics that could be utilized in the development and reporting of HSG to optimize quality. Process principles also depict the subjective and/or objective belief systems in place that represent the preferences (what ought to be) of individuals, groups, or populations on the course(s) of action for addressing a health systems challenge. Process principles also refer to how the guidance is made public in a way that is consistent and comparative as this can facilitate their comprehension, which will further enhance their uptake and aid in easing their application. Therefore, when developing, appraising, or reporting HSG, a conceptual understanding of the process is fundamental. Table 1 shows concepts for HSG process principles.

**Table 1:** Process principles

1. Prioritization	The guidance fits in properly and is consistent with current health system priority areas within all applicable system levels and sectors by targeting a priority topic/jurisdiction/population [4, 5, 53–59]. The guidance addresses these specific local priority areas with a clearly documented/demonstrated need and also informs policy decisions on how to further prioritize across competing areas [4, 53, 54, 59, 60]. The origin of the mandate to develop the guidance [59] is also reported (for example, guidance that is mandated by a top official like the Minister of Health is considered to be of high priority).
2. Relevance	The guidance recommendations should be relevant to, appropriate to, and valid for the health system issue being addressed and relevant to the target population [5, 55–57, 60–63]. The recommendations are relevant to the setting within which the guidance will operate, the institutional needs of that system/sub-system, as well as local, national, and potentially global needs [54, 64–68].
3. Timeliness	The recommendations are available in a timely manner in relation to when the policy decisions are made or timely in relation to the health system issue being addressed [55, 56, 61, 69, 70]. The guidance is timely and usable by the broad range of health systems stakeholders [53, 68, 71] since some policy decisions are sometimes made within crucial corresponding time frames or as windows of opportunity open and close [4, 62].
4. Scope	The guidance is comprehensive and covers all relevant/appropriate (direct and indirect) health system levels, sub-systems, and sectors [5, 8, 56, 61, 63, 71, 72]. This also includes the various relevant sub-systems/components (hospitals, regional health authorities, and public health units etc.) within the health system. Identifying the scope is important because these various components are interlinked, interdependent, and interact at various interfaces for overall health system performance [53, 55, 58–60, 71–74].
5. Transparency	Systematic, replicable, and transparent processes are applied in developing and reporting the guidance [52, 61, 75, 76]. These processes are systematic and transparent enough for the methods of development/reporting of the guidance to be reproducible [59, 74]. In order to paint a clear picture to knowledge users and target populations, sufficient details on these processes are provided [5, 54, 63, 65, 67].
6. Evidence- based	The best available research evidence informs the recommendations [4, 5, 54, 55, 57, 58, 60, 61, 63, 69–71, 73–77]. The type(s) of evidence that was used to generate the guidance is/are stated, and this can range from well-established scientific methodologies or it can also be non-experimental (for example, colloquial evidence, anecdotal evidence, or preliminary models) [8, 62, 63, 70, 75, 78]. The evidence is context sensitive enough to resonate with local realities [4, 5, 54, 56, 57, 60, 61, 63, 69–71, 74, 75].
7. Stakeholder involvement	Alternative views on the policy issue and the complementary expertise of a multidisciplinary group of relevant stakeholders are considered in the development of the guidance [4, 5, 8, 53–55, 58–64, 67–69, 71, 75–77, 79]. Guidance developers, those involved in the implementation and evaluation of the guidance, and those who will be affected by the guidance recommendations are involved in the development process [54, 56, 57, 66, 71, 74, 80].
8. Ethical	The recommendations reflect considerations of an ethical lens [4, 8, 54, 56, 76] and align with applicable ethical principles and values (for example, equity, equality, human rights, liberty, efficiency, autonomy, dignity, beneficence) [8, 55, 63, 65, 74, 80, 81]. The guidance adequately promotes fairness and equality in terms of age, ability, culture, gender, socioeconomic status, religion, occupation, language, ethnicity, race, or sexual orientation among the target population [60, 61, 66, 69, 79, 82].
9. Outcomes	The guidance describes all the anticipated effects/outcomes as well as the appropriate indicators that can be used to measure the effects/outcomes [8, 54, 57, 58, 66, 67, 74, 75]. Adequate rationale regarding the choice of the outcomes and the indicators selected is provided [8, 58]. Considering potential uncertainties that may result, alternative outcomes and outcome indicators are also identified. Performance thresholds, targets, and standards that are considered acceptable are also identified [8, 58, 65–67, 76].
10. Competing interests	A declaration of competing interests (for example, financial, academic, professional) by the guidance developers, whether direct or indirect, is/are made in advance [57, 74, 80]. The author’s positions, roles, and affiliations are clearly stated [65]. Any reported or identified conflicts of interest are managed, with a description of the approaches used to curb any influence clearly documented [57, 80]. It is also clear that the views of any funding body involved have not influenced the development process of the guidance [65].
11. Presentation	The recommendations are clear, succinct, unambiguous, and presented in a readable and consistent format [57, 60, 76, 81], with key recommendations easily identifiable [53, 57, 58, 66]. The guidance is presented in a manner that is uniform, user-friendly, and easy to navigate [4, 53, 61, 64, 68, 80]. It contains an executive summary, full text, a complete list of relevant references, a glossary of terms, and full meaning of abbreviations and contact information of authors. Words or phrases denote an aspirational rather than a mandatory intent [80].

We were able to point out patterns and draw linkages between the process principles concepts. We offer five examples here. First, guidance should be relevant to and developed for health system areas with a clearly demonstrated and documented need, and the feedback of appropriate stakeholders will further highlight priority areas, which may potentially lead to timely interventions. Second, systematic and transparent approaches have to be applied to search for and identify relevant evidence, which should also be available in a timely manner. Third, ensuring that stakeholders from all applicable health system and sub-system levels are involved in the HSG process, that relevant evidence is sought and that appropriate outcomes are chosen can enhance the comprehensiveness of the guidance recommendations. Fourth, having clearly defined and consistent outcomes and declaring and managing interests as well as engaging in a participatory approach that incorporates the various perspectives of multiple stakeholders is also important for transparency of HSG. Finally, the ethical lens applied will be impacted by the quality of the evidence available, the composition of the involved stakeholders, the outcomes/indicators selected, the health system levels/sector involved, and whether it is a priority area that is relevant to the setting.

#### Content

Content (the “what” of the HSG) represents the topics, subjects, and substance that demonstrate the content integrity of the guidance recommendations [44, 46]. Here, we find statements about the impetus of the endeavor that provides direction on the HSG objectives and goals. Keeping in mind that in the absence of an appropriate economic lens, even the best-designed HSG may not achieve adequate success; we also find here the myriad of economic factors that come with the HSG process and need to be taken into account. In addition, within the content of the HSG, it is also important to consider and reflect on the fact that not all guidance recommendations go into practice as planned and events may occur that may derail intended actions. We also find here assessment considerations, usually ongoing throughout the HSG process, and which refer to those elements that assist in determining whether the guidance process was properly followed and/or records the impact/outcomes of the HSG. The content of the HSG also articulates the operationalization of the proposed recommendations and carries information on how to best reach the target users of the guidance. Table 2 shows concepts for HSG content.

**Table 2:** Content

12. Problem definition	The health systems challenge (for example, financial, governance, or delivery arrangements) and its causes are clearly articulated (including any links/integration with other policy problems on the government’s agenda) [5, 75, 83]. The nature, causes, magnitude, frequency, and intensity of the problem, the populations, and jurisdictions that are affected are clearly described [57, 60, 62, 64, 66, 71]. Appropriate rationale exists to justify that either new guidance is needed or existing guidance of acceptable quality can be adapted and used to address the problem [4, 56].
13. Operationalization	The recommended “solutions” are operationalized sufficiently with the conceptualization, operational guidance, and the mode of delivery of the options clearly stated [8, 54, 60]. For example, the guidance provides instructional support for their successful operation and staff training that corresponds with the guidance expectations. Training recommendations could be in the form of a course, a workshop, accompanying manuals, or consultancy services that staff can refer to during the implementation phase in order to standardize practice [8, 54, 58, 60, 61, 63, 73, 74]. If technical assistance (research institutes, consulting firms, NGOs) is required, this is identified and documented [8].
14. Costs	The guidance clearly documents a tentative budget required to implement the guidance recommendations [8, 64, 65, 74, 77, 84]. The potential financial costs (including downstream costs) of the operation are stated so that decision makers can assess the feasibility of the guidance implementation and evaluate whether the cost of implementing the guidance will be worth its potential impacts [5, 64, 73, 74].
15. Resources	The inputs and resources required to implement the recommendations are clearly defined and they have to be proportionate to the health system problem that is being addressed [55, 57, 61, 71, 73, 79]. Some of these resources could be time, infrastructure, administrative capacity, information, equipment, supplies, healthcare professionals, training etc. [55, 56, 58, 59, 61, 69, 76]. The guidance provides a description of the amount, frequency, and duration of the inputs and resources required [54, 57, 71].
16. Effectiveness	The guidance reports whether the anticipated goals and objectives have been achieved elsewhere or in a similar setting/condition, either through evidence from evaluation studies done at other sites (if available) or from expert opinion [54–56, 69]. In describing this effectiveness, the guidance makes projections on how and why the objectives and goals will be achieved in the current setting [64, 69, 75].
17. Cost-effectiveness	The recommendations are attentive to value for money considerations [57, 63, 84]. Sound local or applicable evidence (wherever available) on the cost-effectiveness of the guidance recommendations are provided [54, 57, 60, 63, 79]. These traditionally report costs, direct and indirect program inputs/resources, and outcomes to guide health policy decisions and provide benchmark(s) or threshold(s) that the health system is willing to accept or support in relation to other competing health system priorities [54, 60, 73].
18. Benefits/harms	Description of the potential unintended consequences (positive and negative) of the guidance is provided or an assessment/judgment of the potential benefits/harms are made [60, 67, 75]. Descriptions of the populations or institutions that may experience significant impacts are identified [57, 60, 65].
19. Dissemination plan	Strategies for communicating the guidance are included with a clear dissemination framework, the mode of delivery, and the integrity of the avenue used for dissemination been properly reported [54, 65, 81]. The proposed strategies for disseminating the guidance are tailored to the relevant audiences (for example, a formal written report, user-friendly summary, oral presentation, poster, press release, booklet, workbook, films, pocket card) [57, 74].
20. Process evaluation	This involves recommendations for evaluating the structure and process of implementation as well as corresponding challenges [61, 68, 80]. This evaluation examines the extent to which the guidance recommendations were implemented as planned, and also provides a way to monitor the process and make adjustments and improvements to implementation strategies [74, 79]. It documents the inputs, services, and activities that were implemented, and can identify potential strengths, weaknesses, opportunities, and threats to the implementation process [62, 79].
21. Outcomes/impact evaluation	An assessment of the outcome/impact of the guidance is recommended to determine whether the course of action was a success or failure. There are recommendations on measuring the results, or outcomes of the guidance in a way that determines whether the changes observed in relation to the health system challenge being addressed can be attributed to the guidance [58, 61, 80, 82]. There are also recommendations for an impact evaluation to look at the short- and long-term deeper primary and secondary changes that resulted from the guidance [57, 69, 74, 77].
22. Updating	Recommendations for periodic updates are made and the procedure to update the guidance is provided with explicit timelines on anticipated review [57, 68], appropriate expiration date of the guidance, and an explanation of the rational for the proposed time frames [80]. Setting time frames for periodic updates ensures that guidance producers revisit the recommendations and respond accordingly to potential health system changes and emerging challenges. Also, the recommendations should be current, and the evidence (for example, systematic reviews) on which they are based is considered recent and up-to-date [76, 80].

As with the process principles, we were also able to point out patterns and draw linkages between the content concepts. For example, the way the problem is defined can provide direction on the costs and resources required for implementation of the solution, reveal some potential unintended consequences, and guide the operational plan. The operational plan can also provide hints on some potential unintended consequences. A clear problem definition will also inform the process and outcomes/impact evaluation of the HSG. Designing the operational options can inform the costs and resources required. The costs, resources, and operational plan will in turn influence whether the HSG will be effective and/or cost-effective. The updating plan is also contingent on the health system issue being addressed, the effectiveness of the guidance recommendation, and the resources required.

#### Context principles

Context principles represent those systemic factors like the local technical, situational, structural, institutional political, and socio-cultural components of the health policy environment that can impact HSG recommendations [44, 46]. Like health policymaking, HSG does not occur in a vacuum so it is important to pay attention to the variety of contextual factors that may have significant impact on how the guidance is developed, adapted, and implemented by the end users [48, 49]. The process principles and the content of the HSG all have to be contextualized; therefore, context principles impact these other clusters of the concepts. Context principles refer to the usability in context, and here, we find concepts related to those system components that make up the setting within which the HSG is to be used. A clear overview of the context or setting that will be impacted by the guidance recommendations is essential. This will provide an understanding of why some guidance recommendations may work in some settings and not others. We find here factors that will enhance and facilitate the adherence to HSG recommendations as per protocol. We also find here factors that represent the values and moral fabric of the society and how this can either facilitate or impede the HSG recommendations. Table 3 shows concepts for HSG context principles.

**Table 3:** Context principles

23. Feasibility	The guidance recommendations are realistic and the actions are pragmatic [4, 5, 53, 55, 57, 59, 61, 63, 68, 70, 81, 84]. The guidance describes facilitators and barriers for implementation [58, 69]. It is clearly demonstrated that the implementation of the guidance is feasible within the proposed practice environment, and the recommendations match local capacities and expectations [4, 8, 53, 61, 63, 69, 71, 80].
24. Affordability	The guidance recommendations are affordable within the financial structure and budgetary allocations of the health system [53, 54, 56, 69]. Potential sources of local government funding and donor organizations are identified. For policy issues in which there may be several sources of funding, the guidance also considers the level of coordination among the donors and between the donors and the local government [56, 58, 59, 61, 69].
25. Flexibility	The guidance is flexible and adaptable to the expertise of the user and the varying local conditions. It acknowledges the importance of professional judgment and discretion and provides recommendations that users can adapt in accordance with their own individual circumstances and needs [57, 71, 80]. The recommendations steer away from the adoption of rigid approaches so as not to inappropriately or unnecessarily limit those in charge of applying them [57, 71].
26. Socio- culturally acceptable	Considering the diversity of values in many regions, the recommendations are robust under societal and cultural scrutiny by adopting a socio-cultural perspective [54, 57, 61–63, 69, 73]. It recognizes socio-cultural expectations and provides an understanding of the role that socio-cultural factors will play in the success of the guidance recommendations [54, 55, 69, 71, 73].
27. Politically sound	The political acceptability of the recommendations is considered in order to assess if they align with political interests/commitments [54, 57, 63, 64, 70, 75, 79, 81, 82]. Implementation of guidance can stir swings in the national mood, lead to changes in the balance of organized forces, such as interest groups, or influence outcome of events within the government, for instance an election [85]. Therefore, options proposed that are in sync with the political climate may garner adequate support from top policy/government officials [58, 73].
28. External factors	Determinants of health system performance that lie outside the formal architecture of the health system but will influence the performance of its functions are considered; for example, judicial system, social system, recession, corruption, state of the economy [48, 54, 73, 79]. These are non-health system factors originating from other local institutional organizations that impact on the usual operations of the health system [54, 59, 64, 80].
29. Generalizability	The recommendations are transferable to other settings with similar health system features; for example other countries or regions [59, 65, 66, 79]. Judgments are made about the applicability of the recommendations beyond its original context (setting or population) to ensure that contexts with similar institutional, socioeconomic, and political demographics facing an identical health system challenges can adapt and use the guidance [55, 60, 65, 74].
30. Sustainability	The guidance provides an indication of the sustainability of the effects of the recommendations to show that long-term outcomes can be continuously achieved and maintained at an acceptable level [56, 61, 64, 69]. Due to constantly evolving health system issues, looming budget cuts, fluctuating resources, rising costs of new technologies, an ageing population, shifting burdens of diseases etc., it is crucial to develop recommendations that will stand the test of time [56, 60, 69].

We were able to point out patterns and draw linkages between the context principle concepts as well. We offer some examples here. For HSG to be feasible and sustainable, it should be affordable and resonate with local values (political and socio-cultural). Information on affordability, feasibility, and sustainability may also determine whether the guidance recommendations can be transferable to other settings. Providing socio-culturally appropriate recommendations may also indicate which other health systems can adapt and use the HSG. Also, the HSG should be flexible enough to accommodate shifts in values (e.g., political, socio-cultural). Additionally, HSG that reflects the socio-cultural preferences of the target population, that is affordable, feasible, sustainable, and transferable to comparable settings, can amass support from politicians. The external factors that originate from other institutional systems may impact the feasibility and sustainability of the guidance and may be influenced by the political and socio-cultural climate.

### Synthesis across domains

**Fig. 2:**
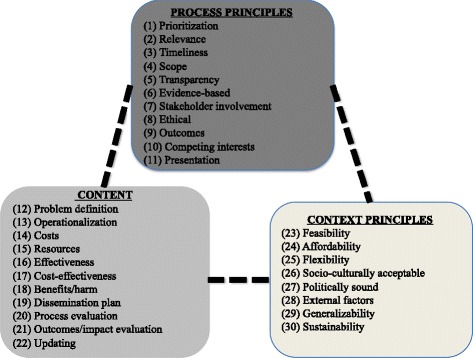
Framework of health systems guidance concepts

As is expected of a Critical Interpretive Synthesis (CIS), we were able to point out relationships between the concepts across the three clusters as shown in Fig. 2. We highlight some relationships:

- ➢ Uptake of guidance can be enhanced if it is addressing a priority area for which evidence-based reports of effectiveness and cost-effectiveness exist.
- ➢ Consultations with appropriate stakeholders are also crucial for feasibility of implementation and sustainability of the HSG because sometimes, the individuals tasked with implementing guidance recommendations may not be committed to them and this may influence adherence. Their input into the HSG process and support for the recommendations may alleviate this concern.
- ➢ HSG outcome(s) chosen will influence the way the problem is defined and provides information that will be useful for evaluating the HSG.
- ➢ Including an ethical viewpoint will also impact how the problem is defined and will influence cost-effectiveness thresholds.
- ➢ Systematic and transparent processes may provide an impetus for donor involvement (affordability).
- ➢ Information on affordability can determine whether the health system issues can be addressed in a timely manner and inform judgments on how to evaluate the process.
- ➢ The HSG should be flexible enough to accommodate constantly evolving evidence and changing health system priorities.
- ➢ The guidance should provide socio-culturally appropriate solutions that are relevant to the applicable levels/sectors of the health system.
- ➢ HSG that is in line with ethical principles, addresses priority issues, and is timely will be appealing to politicians.
- ➢ The external alignment of guidance may affect the effectiveness and impact the operational considerations.
- ➢ The outcomes selected, the system level/sectors involved, or the ethical values in place may expose the external factors that may be pertinent.
- ➢ Some determinants of generalizability of the guidance are the stakeholders involved, the evidence used, transparency of the process, ethical lens considered, and outcome/indicators selected

## Conclusions

This project is the first phase of a multistage approach to create an internationally useful HSG tool, AGREE for Health Systems (AGREE-HS) that will inform the development, reporting, and appraisal of HSG. Modeling after the paradigm to create a tool for clinical practice guidelines [50], our first step was to conduct a review of the published and grey literatures to identify concepts related to HSG quality. In this vein, it was our expectation that the receptiveness, adoption, and diffusion of HSG recommendations depend on the perception of their quality, and with this study, we aimed to identify those core components of good quality HSG.

We found a total of 30 potential HSG appraisal concepts that have the capacity to discriminate between high- and low-quality guidance and direct their development and reporting. We found no existing tools to support HSG appraisal and found few studies describing concepts that were directly tested to appraise HSG. Indeed, the papers we examined reflected a variety of study designs and goals; none reflected methods used to develop appraisal methods. However, the data from our studies show a convergence of ideas in the HSG research community about what constitutes good and useable HSG. Together, these data can provide the foundation of a tool that guides HSG developers in the types of information important to report in a HSG document and tactics for optimal execution. These concepts also address issues of appropriateness and completeness as well as information, which are important components in the uptake of guidance recommendations.

One strength of this study is that we used a sound knowledge synthesis strategy, the critical interpretive synthesis (CIS) approach. We acknowledged the paucity of data related to HSG appraisal, as well as the diversity in the literature sources and types of available data. Also, given that HSG is not a highly bounded topic, we did not rely on a narrow initial research question; instead, we were refining it as the review progressed. The various methodological stages did not proceed as discrete entities as there was a constant to-ing and fro-ing throughout the review and synthesis process; at times, we were concurrently searching, sampling, critiquing, and analyzing. The CIS approach is both systematic and iterative with an interpretive approach to analysis and synthesis of data that allowed us to capture and critically analyze an in-depth depiction of how to differentiate between HSG on the basis of quality, among other considerations. Through CIS methodology, we were able to further identify and include additional papers that were not directly related to HSG but made valuable theoretical contributions to the process of appraising guidance [20, 27].

A weakness of our study is in the nature of the CIS strategy, which exposes us to the risk that certain concepts may have been missed (calling saturation too soon, for example). If we have missed important elements, we anticipate they will be identified in the second stage of our research. Also, the health policy analysis triangle framework is made up of four elements: process, content, context, and actors. But for our analysis, the original framework was modified as we included “actors” in the process principles. Actors (the who of policy) refer to local, national, or international individuals or groups (governmental and non-governmental) involved in the policy process [44, 45]. We found that actors could be incorporated under the process principles concept “participatory.” Additionally and at first glance, it appears that there is the risk that some concepts may lead to potential contradictions. For example, in some jurisdictions, achieving an ethical HSG may not be aligned with achieving a feasible HSG (e.g., in contexts where there is a bias toward citizens because of race or sexual orientation). Currently, the data provides no guidance on how to reconcile a situation like this.

In our analysis, we showed the role that the process principles, content, and context principles plays in the development, appraisal, and reporting of HSG and demonstrated the link and resonance within and between their various concepts. We pointed out patterns and drew linkages between the concepts in order to show that the concepts and domains do not typically occur in a linear, independent, or discrete manner. The interaction between these three clusters is an imperative consideration because they all influence the guidance process and can facilitate or impede the success of HSG recommendations.

Some of the concepts (criteria or items) and domains identified in this review may not be applicable to every jurisdiction or country so they are not intended to serve as a blueprint for all health systems to strictly apply. Guidance documents are administrative instruments that provide recommendations and implementation options typically in a step-by-step format but do not have force of law and, as such, allow for accommodations in approach [51, 52]. They are intended to provide recommendations on how to comply with governing statutes and regulations and assist staff and managers on how institutional mandates and objectives should be implemented in a manner that is fair, consistent, and effective [52]. Therefore, development of high-quality HSG will impact the type of recommendations being formulated, the degree to which they get implemented, the methods of dissemination, and the extent to which they impact on the usual operations of the health system [51].

Different players in the HSG process play different roles at different times and under different circumstances. Developers of guidance may only be looking at process principles and content, while end users in the field may focus more on usability of the guidance in their individual contexts. Thus, for example, to optimize this dual perspective and facilitate the division of labor, there is interest at the WHO to produce workbooks where the global guidance they develop (process principles and content) will be complemented by having a companion workbook for those at the receiving end (context principles). In this case, the role of the WHO will be the development of global guidance, while the country’s role (or health system’s role) will be to take this global guidance and then apply it to their local context. This makes our framework of health systems guidance concepts quite practical.

Our study has elicited 30 unique concepts. It is unclear the extent to which HSG stakeholders will view all concepts as equally important to our proposed tool, or, moreover, given the potential roles of the tool (development, appraisal, reporting), if there are varying levels of priority as a function of purpose. The next phase (stage 2) of our research program is aimed to create the beta version of the AGREE-HS by having international stakeholders prioritize these concepts and using those results to populate the tool. Our final phase (stage 3) will involve the usability testing of the beta version of the tool.

## Appendix

**Table 4:** Summary of selected papers

Author(s)	Title	Organization/location^a^	Purpose of the study and relevance of its contribution	Concepts extracted
Murray and Frenk [72]	*A WHO framework for health system performance assessment*	WHO/Switzerland	This paper discusses how variations in health outcomes across different countries are related to differences in health system performance (design, content, and management of health systems) and proposes a framework to assess and advance the understanding of health system performance.	Timeliness; scope; evidence-based; stakeholder involvement; ethical; outcomes; operationalization; costs; feasibility; socio-culturally acceptable; politically sound; external factors
German et al. [71]	*Updated guidelines for evaluating public health surveillance systems*	CDC/USA	The paper aims to provide an operational framework and guidelines for evaluating the quality, efficiency, and usefulness of a public health surveillance systems.	Timeliness; scope; evidence-based; stakeholder involvement; problem definition; resources; outcomes/impact evaluation; feasibility; socio-culturally acceptable; generalizability; sustainability
Davies and Littlejohns [53]	*Views of directors of public health about NICE appraisal guidance: framework and guidelines for evaluating results of a postal survey*	NICE/UK	The aim is to explore the view of Directors of Public Health with regards to the development, implementation, and dissemination of appraisal guidance for health technologies within the UK health system.	Prioritization; timeliness; scope; evidence-based; stakeholder involvement; presentation; feasibility; affordability; socio-culturally acceptable; politically sound
APA [80]	*Criteria for practice guideline development and evaluation*	American Psychological Association / USA	The paper is designed to promote quality and consistency in practice guideline development and to describe the criteria by which practice guidelines are developed, evaluated and reviewed.	Prioritization; relevance; transparency; evidence-based; stakeholder involvement; ethical; outcomes/impact evaluation; presentation; feasibility; flexibility; external factors
Shaw and Kalo [54]	*A background for national quality policies in health systems*	WHO / Switzerland	The paper aims to outline some of the values, forms, and concepts which affect national approaches for the improvement of quality as a central element for reform of health systems and health service delivery.	Prioritization; transparency; evidence-based; stakeholder involvement; ethical; operationalization; resources; effectiveness; dissemination plan; affordability; socio-culturally acceptable; politically sound; external factors
Wilson [81]	*How to find the good and avoid the bad or ugly: a short guide to tools for rating quality of health information on the internet*	European Commission / Belgium	The aim is to report methods, a set of criteria for good practice and tools for evaluating and rating the quality of health information.	Ethical; feasibility
Arah et al. [55]	*Conceptual frameworks for health systems performance: a quest for effectiveness, quality, and improvement*	University of Amsterdam / Nederlands	The aims are to understand the underlying concepts of national and international performance frameworks for health systems (case studies: UK, Canada, Australia, the USA, the WHO, and the OECD); to explore health system efficiency and performance indicators; and examine how and in what context the resultant performance data can be used to drive improvement.	Prioritization; relevance; timeliness; scope; evidence-based; stakeholder involvement; ethical; outcomes; resources; effectiveness; outcomes/impact evaluation; feasibility; affordability; socio-culturally acceptable; politically sound; generalizability
Murray and Evans [86]	*Health systems performance assessment: debates, methods and empiricism*	WHO / Switzerland	The aim is to explore the role that the WHO plays in providing advice to member states on how best to organize, manage, and strengthen their health systems. It discusses how recommendations for clinical practice decisions differ from health system recommendations.	Outcomes; evidence-based
Travis et al. [87]	*Overcoming health-systems constraints to achieve the millennium development goals*	WHO / Switzerland	The paper uses the Millennium Development Goals as a reference point to explore the advantages and disadvantages of approaches to health system strengthening through the lens of individual service or disease specific initiatives.	Prioritization; evidence-based; stakeholder involvement; outcomes; operationalization; resources; cost-effectiveness; feasibility; affordability; external factors; generalizability; sustainability
Lomas et al. [78]	*Conceptualizing and combining evidence for health system guidance*	Canadian Health Services Research Foundation / Canada	This review examines how guidance developers and policy-makers view evidence as well as how different forms of evidence can be combined to produce health system guidance.	Relevance; transparency; evidence-based; stakeholder involvement; outcomes; ethical; problem definition; effectiveness; feasibility; flexibility
Arah et al. [64]	*A conceptual framework for the OECD health care quality indicators project*	University of Amsterdam / Nederlands	The aim is to provide a sound conceptual framework that defines what is meant by quality of health care and to place it within a wider performance framework which acknowledges the key health policy goals adopted by the OECD and its member countries as they formally assess and “incentivize” the performance of their health care systems.	Relevance; timeliness; stakeholder involvement; ethical; problem definition; cost; effectiveness; feasibility; affordability; socio-culturally acceptable; politically sound; external factors; sustainability
Oxman et al. [65]	*Improving the use of research evidence in guideline development: reporting guidelines*	Norwegian Knowledge Centre for the Health Services / Norway	The aim is explore the standard formats for wide variety of WHO policies, recommendations or guidelines and how these recommendations should be formulated and reported. It emphasizes that the information needed to judge the quality of guidance and determine its applicability and adaptability should be reported.	Relevance; timeliness; transparency; evidence-based; ethical; presentation; outcomes; problem definition; operationalization; cost-effectiveness; outcomes/impact evaluation; dissemination plan; affordability; generalizability
Schünemann et al. [88]	*Improving the use of research evidence in guideline development: 1. guidelines for guidelines*	McMaster University / Canada	This report from the WHO Advisory Committee on Health Research is aimed at providing advice to the WHO on the use of more rigorous processes to ensure that the best available research evidence informs health care recommendations.	Prioritization; evidence-based; stakeholder involvement; ethical; outcomes; competing interests; presentation; problem definition; operationalization; costs; benefits/harm; process evaluation; outcomes/impact evaluation; updating; flexibility; generalizability
Islam [56]	*Health systems assessment approach: a how-to manual*	USAID / USA	The aim of this report is to enable USAID Missions to assess a country’s health system during early phases of program development or sector planning. Using a performance indicator-based and health indices approach, the report is designed to provide a rapid and comprehensive assessment of key health systems functions (Governance, Health financing, Health service delivery, Human resources, Pharmaceutical management, Health information systems) and propose recommendations for improvement.	Prioritization; relevance; timeliness; scope; evidence-based; stakeholder involvement; ethical; resources; effectiveness; affordability; sustainability
National Health and Medical Research Council (NHMRC) [57]	*Standards and procedures for externally developed guidelines*	Australian Government / Australia	The aims is to inform external persons and organizations of the procedures and key steps to be followed in developing, implementing, and evaluating guidelines that are intended for submission to the NHMRC for approval.	Prioritization; relevance; timeliness; evidence-based; stakeholder involvement; outcomes; presentation; problem definition; resources; effectiveness; cost-effectiveness; outcomes/impact evaluation; dissemination plan; feasibility; flexibility; socio-culturally acceptable; politically sound
Øvretveit and Klazinga [61]	*Guidance on developing quality and safety strategies with a health system approach*	WHO / Denmark	The aim is to provide tools and approaches to help national policy advisers and policy-makers to create and implement a national quality strategy, drawing attention to the need for sustainable longer term public health measures in order to improve their health systems and engage member states in a constructive dialogue.	Prioritization; relevance; timeliness; scope; transparency; evidence-based; stakeholder involvement; ethical; presentation; operationalization; resources; process evaluation; outcomes/impact evaluation; feasibility; affordability; socio-culturally acceptable; politically sound; sustainability
Van der Sluijs et al. [62]	*Exploring the quality of evidence for complex and contested policy decisions*	Utrecht University / The Nederlands	The aim is to provide a deeper understanding and increased awareness of the phenomenon of uncertainty and its policy implications, by discussing some key quality aspects of knowledge production and use especially in complex policy issues.	Relevance; timeliness; evidence-based; stakeholder involvement; outcomes; problem definition; process evaluation; outcomes/impact evaluation; socio-culturally acceptable
Hoffman et al. [83]	*The use of research evidence in two international organizations’ recommendations about health systems*	McMaster University / Canada	This study systematically compares health systems recommendations by international organizations (WHO and the World Bank) to the research evidence that was available at the time of their formulation. These recommendations about health systems (on technical guidance for example) have the potential to link research to action by acting as mediators between the best available research evidence and policy options.	Prioritization; relevance; transparency; evidence-based; stakeholder involvement; ethical; outcomes/impact evaluation; feasibility; affordability; politically sound
NHS [60]	*How to use NICE guidance to commission high-quality services*	NHS / UK	The guide is aimed for people involved in commissioning health and social care services and public health programs in the UK and provides guidance that can help to support the commissioning of new productive, efficient, and high-quality services, provides an implementation tool for planning and prioritizing services, and a framework for the evaluation or redesign of existing services and the decommissioning of ineffective interventions.	Prioritization; relevance; scope; evidence-based; stakeholder involvement; ethical; presentation; problem definition; operationalization; cost-effectiveness; outcomes/impact evaluation; generalizability; sustainability
NHS [66]	*Methods for the development of NICE public health guidance*	NHS / UK	The paper describes the philosophical and methodological principles which govern the production of guidance for public health practice by NICE and the key components involved.	Relevance; stakeholder involvement; ethical; outcomes; presentation; problem definition; generalizability
Oxman et al. [75]	*SUPPORT tools for evidence-informed health policymaking: what is evidence-informed policymaking?*	Norwegian Knowledge Centre for the Health Services / Norway	This article focuses on how to better use research evidence (what constitutes evidence? What is its role in health policy?) to inform decisions about how best to organize health systems, including arrangements for delivering, financing and governing health services, and strategies for bringing about change.	Prioritization; transparency; evidence-based; stakeholder involvement; ethical; outcomes; benefits/harms; politically sound
Perpiñán et al. [84]	*Quality assessment of economic evaluations in health care: a checklist and user guide*	Murcia University / Spain	The aim is to promote the efficiency in the process of incorporating new health technologies, as well as to guide their implementation in health systems by reporting an instrument composed of a user guide and a 12-criteria checklist in which a score is assigned to each items.	Problem definition; cost; feasibility; socio-culturally acceptable; politically sound
Savigny and Adam [89]	*Systems thinking for health systems strengthening*	Alliance for Health Policy and Systems Research, WHO	This report offers a practical systems thinking approach to decipher the complexity of health systems, identify health systems challenges, and then applies that understanding to design better interventions to strengthen health systems and improve health.	Scope; relevance; transparency; evidence-based; stakeholder involvement; ethical; outcomes; problem definition; cost; resources; effectiveness; cost-effectiveness; benefits/harms; process evaluation; outcomes/impact evaluation; feasibility; affordability; politically sound
WHO [69]	*Practical guidance for scaling up health service innovations*	WHO / Switzerland	The aim is to identify general principles and make specific suggestions on the process of scaling up successfully tested health services innovations and discusses the strategic choices that facilitate and hinder the process.	Timeliness; evidence-based; stakeholder involvement; ethical; operationalization; resources; effectiveness; outcomes/impact evaluation; feasibility; affordability; socio-culturally acceptable; politically sound; sustainability
Moher et al. [76]	*Guidance for developers of health research reporting guidelines*	Faculty of Medicine / University of Ottawa	The aim is to update and expand upon efforts to outline a strategy for developing reporting guidelines and shows that reporting guidelines is associated with improvements in the quality of reporting health research. An 18-step checklist on how to develop a reporting guideline is provided.	Transparency; evidence-based; stakeholder involvement; presentation; problem definition; resources; dissemination plan
Swanson et al. [90]	*Toward a consensus on guiding principles for health systems strengthening*	Brigham Young University / USA	The paper proposes a list of ten guiding principles necessary for the development and communication of clear and consistent frameworks for policy, practice, and evaluation with the overall goal of strengthening health system.	Scope; transparency; evidence-based; stakeholder involvement; ethical; problem definition; operationalization; effectiveness; cost-effectiveness; outcomes/impact evaluation; affordability; socio-culturally acceptable; politically sound
Etienne et al. [91]	*Health systems financing: the path to universal coverage*	WHO / Switzerland	This report provides practical guidance on ways to finance health care by transforming available evidence-based practices into a menu of options for raising sufficient resources and removing financial barriers to access. Emphasis is placed on moving toward universal coverage to optimize health service provision.	Relevance; scope; evidence-based; ethical; outcomes; feasibility; operationalization; flexibility; timeliness; cost; resources; effectiveness; cost-effectiveness; outcomes/impact evaluation; affordability; politically sound; external factors
German BACKUP Initiative (GIZ) [82]	*Guidance on integrating gender-specific issues into health systems strengthening activities*	Federal Ministry of Economic Cooperation and Development / Germany	The aim is to advise and assist organizations that are planning to apply to the German BACKUP Initiative for technical support on how to analyze and integrate gender-related issues into Health Systems Strengthening (HSS) activities. A checklist used to plan for technical support and develop applications that take into consideration specific gender dimensions in the different components of a health system is provided.	Ethical; stakeholder involvement; socio-culturally acceptable; politically sound
Sheikh et al. [92]	*Building the field of health policy and systems research: framing the questions*	Public Health Foundation of India / India	This paper discusses the state-of-the-art in Health Policy and Systems Research (HPSR), addresses the current challenges and opportunities for the field, and lays out what is needed to build capacity in HPSR and support local policy development and health systems strengthening.	Prioritization; relevance; scope; stakeholder involvement; outcomes; problem definition; operationalization; feasibility; flexibility; socio-culturally acceptable; politically sound; generalizability
WHO [11]	*Health system strengthening: improving support to policy dialogue around national health policies, strategies and plans*	WHO / Switzerland	This report reviews experiences with conducting and supporting policy dialogue for the development or renewal of comprehensive policies, strategies, and plans to improve health service delivery, health outcomes and strengthen health systems.	Prioritization; relevance; scope; evidence-based; stakeholder involvement; outcomes; operationalization; costs; resources; effectiveness; feasibility; affordability; politically sound; external factors; generalizability; sustainability
Atun [73]	*Health systems, systems thinking and innovation*	Faculty of Medicine, London Imperial College / UK	The aim is to discuss factors that influence the achievement of health system performance and efficiency and provide an understanding of why many well-intentioned policies and managerial decisions aimed at improving health systems do not achieve desired outcomes but lead to unexpected or unintended consequence.	Scope; evidence-based; ethical; problem definition; operationalization; costs; resources; effectiveness; feasibility; affordability; socio-culturally acceptable; politically sound; external factors
Bosch-Capblanch and Allen [70]	*Health systems strengthening and conflict: transformation in fragile states*	Tropical and Public Health Institute / Switzerland	The aim is to inform programming, policy, advocacy, research, and the civil society on the best ways to strengthen health systems. Main challenges encountered in producing methods to develop health systems guidance are discussed.	Timeliness; evidence-based; costs; effectiveness; feasibility; politically sound
Bosch-Capblanch et al. [4]	*Guidance for evidence-informed policies about health systems: rationale for and challenges of guidance development*	Tropical and Public Health Institute / Switzerland	The aim is to assess extent to which the need for health systems guidance is part of national policies and plans and assess how guidance is currently formulated. Conceptual and the methodological challenges in the development and use of health systems guidance and ways to address them are discussed.	Prioritization; relevance; timeliness; evidence-based; stakeholder involvement; ethical; outcomes; presentation; problem definition; cost-effectiveness; outcomes/impact evaluation; dissemination plan; feasibility; external factors
Lavis et al. [5]	*Guidance for evidence-informed policies about health systems: linking guidance development to policy development*	McMaster University / Canada	The aim is to discuss the importance of contextual factors in shaping decisions about health systems and discusses the need to work through all the pros and cons of different options before adopting specific health systems guidance. It also shows the need for division of labor between national/global guidance and policy developers to support evidence-informed policymaking about health systems.	Prioritization; relevance; scope; transparency; evidence-based; stakeholder involvement; problem definition; costs; effectiveness; outcomes/impact evaluation; feasibility; politically sound
Lewin et al. [63]	*Guidance for evidence-informed policies about health systems: assessing how much confidence to place in the research evidence*	Norwegian Knowledge Centre for the Health Services / Norway	The aim is to assess how much confidence to place in the types of evidence available on health systems interventions that inform judgments for health systems strengthening. The factors that are important when developing recommendations on policy options regarding health systems interventions are discussed.	Relevance; scope; transparency; evidence-based; stakeholder involvement; ethical; operationalization; cost-effective; feasibility; affordability; socio-culturally acceptable; politically sound
Moga et al. [67]	*Development of a quality appraisal tool for case series studies using a modified Delphi technique*	Institute of Health Economics / Canada	The aim is to outline the process of development of a checklist for quality appraisal of case series studies using a modified Delphi technique. Criteria and items that are useful for appraising the quality of case series are reported.	Relevance; transparency; stakeholder involvement; outcomes; problem definition
Orem et al. [74]	*Do guidelines influence the implementation of health programs? - Uganda’s experience*	WHO / Uganda	The aim is to describe the processes of development, implementation, dissemination, and evaluation of health planning, services management, and clinical guidelines within the health sector in Uganda, with the goal of understanding how these processes facilitate or abate the utility of guidelines.	Prioritization; relevance; scope; evidence-based; stakeholder involvement; ethical; outcomes; problem definition; operationalization; costs; effectiveness; outcomes/impact evaluation; dissemination plan; generalizability
Peters and Bennett [93]	*Better guidance is welcome, but without blinders*	John Hopkins School of Public Health / USA	This paper discusses the challenges related to engendering greater structure and systematization in the development of health system guidance and to the application of evidence to policy.	Evidence-based; stakeholder involvement; benefits/harms; feasibility; flexibility; politically sound; generalizability
WHO [58]	*Guidance on assessing health system building blocks*	WHO / Switzerland	The aim is to provide an overview of the key opportunities and challenges facing a health system (health issues, systemic issues, political/policy issues), and how to address and assess them. It discusses indicators that can be used to assess each of the health systems building blocks and provides a how-to-manual.	Prioritization; scope; evidence-based; stakeholder involvement; outcomes; presentation; operationalization; resources; outcomes/impact evaluation; feasibility; politically sound
Bryce et al. [79]	*A common evaluation framework for the African health initiative*	The Johns Hopkins Bloomberg School of Public Health / USA	The aim is to describe a common evaluation framework for the cross-site initiative to improve population health by strengthening health systems and evaluating the results. Some core elements, inputs, and processes required for strengthening health systems in Africa are discussed.	Stakeholder involvement; ethical; resources; cost-effectiveness; process evaluation; outcomes/impact evaluation; feasibility; politically sound; external factors; generalizability
Philips et al. [77]	*Protocol for development of the guideline for reporting evidence based practice educational interventions and teaching (GREET) statement*	University of South Australia / Australia	The aim is to develop reporting guidelines for evidence-based practice educational interventions and teachings to enable their consistent and transparent reporting in health care. Criteria for appraising practice education for health professional disciplines are provided.	Evidence-based; stakeholder involvement; costs; effectiveness; outcomes/impact evaluation
International Centre for Applied Health Evidence (ICAHE) [68]	The iCAHE guideline checklist	University of South Australia / Australia	The aim is to provide a checklist (items, criteria and domains) to assist in the process of development and appraisal of guidelines.	Relevance; timeliness; evidence-based; stakeholder involvement; presentation; problem definition; feasibility
Funk et al. [59]	*Checklist for evaluating a mental health policy*	WHO / Switzerland	The aim is to explore the processes that are likely to lead to the success of a mental health policy. A framework for the assessment of the quality of the processes and content of mental health policy recommendations is proposed.	Prioritization; scope; transparency; evidence-based; stakeholder involvement; ethical; problem definition; resources; feasibility; affordability; external factors; transferability

^a^Organization/location of the lead authors as reported in the paper

## Abbreviations

AGREE-HS: Appraisal of Guidelines for Research and Evaluation for Health Systems
CDC: Centre for Disease Control
CINAHL: Cumulative Index to Nursing and Allied Health Literature
CIS: Critical Interpretive Synthesis
CPGs: Clinical Practice Guidelines
DAA: Denis Ako-Arrey
EMBASE: Excerpta Medica dataBASE
EVIPNet: Evidence Informed Policy Network
GIN: Guidelines International Network
HSG: health systems guidance
JL: John Lavis
LILACS: Latin American and Caribbean Health Sciences Literature
MB: Melissa Brouwers
MDGs: Millennium Development Goals
MG: Mita Giacomini
NHS: National Health Services
NICE: National Institute of Health and Care Excellence
SA: Saira Akram
USAID: United States Agency for International Development
WHO: World Health Organization
